# Scanning force microscope for *in situ* nanofocused X-ray diffraction studies

**DOI:** 10.1107/S1600577514014532

**Published:** 2014-08-06

**Authors:** Zhe Ren, Francesca Mastropietro, Anton Davydok, Simon Langlais, Marie-Ingrid Richard, Jean-Jacques Furter, Olivier Thomas, Maxime Dupraz, Marc Verdier, Guillaume Beutier, Peter Boesecke, Thomas W. Cornelius

**Affiliations:** a IM2NP (UMR 7334), Aix-Marseille Université, CNRS, Faculté des Sciences, Campus de Saint-Jérôme, Avenue Escadrille Normandie Niemen – Case 142, F-13397 Marseille, France; b Grenoble Institute of Technology and CNRS, BP 75, F-38402 Saint-Martin d’Hères Cedex, France; c European Synchrotron Radiation Facility (ESRF), 6 rue Jules Horowitz, BP 220, 38043 Grenoble, France

**Keywords:** *in situ* atomic force microscopy, nanofocused X-ray diffraction, mechanical properties, nanostructure

## Abstract

A compact scanning force microscope has been developed for *in situ* combination with nanofocused X-ray diffraction techniques at third-generation synchrotron beamlines. Its capabilities are demonstrated on Au nano-islands grown on a sapphire substrate. The new *in situ* device allows for *in situ* imaging the sample topography and the crystallinity by recording simultaneously an atomic force microscope (AFM) image and a scanning X-ray diffraction map of the same area. Moreover, a selected Au island can be mechanically deformed using the AFM tip while monitoring the deformation of the atomic lattice by nanofocused X-ray diffraction. This *in situ* approach gives access to the mechanical behavior of nanomaterials.

## Abstract 2

An atomic force microscope has been developed for combination with sub-micrometer focused X-ray diffraction at synchrotron beamlines and *in situ* mechanical tests on single nanostructures.

## 1. Introduction   

In recent years, studies have shown that nanostructures exhibit mechanical properties which are different from their bulk counterparts. For instance, micrometer and sub-micrometer sized pillars prepared by focused ion beam (FIB) milling revealed an increase of the yield strength with decreasing pillar diameter. This trend became known in the literature as ‘smaller is stronger’ (Minor & Kiener, 2011 ▸; Kiener *et al.*, 2008 ▸; Uchic *et al.*, 2004 ▸). However, micropillars and nanowires which were not fabricated by FIB machining did not show this trend but exhibited strengths close to the ultimate value of the material (Bei *et al.*, 2007 ▸; Richter *et al.*, 2009 ▸). Besides plasticity, elastic properties are also affected by the specimen size as demonstrated for ZnO nanowires which showed an increase of the Young’s modulus with decreasing diameters (Chen *et al.*, 2006 ▸). Recently, reversible phase transitions have been observed for Ni nanowires which were strained up to 34.6%, which is much higher than the typical elastic limit (Wang *et al.*, 2013 ▸). Understanding the aforementioned behaviors for small-scale materials is an important step in mechanical studies and mandatory for the fabrication of future reliable devices based on nanostructures.

In order to shed additional light on the mechanical behavior of low-dimensional materials, *in situ* studies are necessary. In the recent past, several experimental set-ups have been realised for *in situ* mechanical tests of micrometer and sub-micrometer structures in combination with scanning electron microscopy (SEM), transmission electron microscopy (TEM), µLaue diffraction and coherent X-ray diffraction. While SEM is a surface-sensitive tool giving access to slip traces activated during mechanical loading of microstructures and nano­structures (Kiener *et al.*, 2008 ▸; Rabier *et al.*, 2013 ▸; Wheeler & Michler, 2013 ▸), *in situ* TEM studies allow for direct observation of the nucleation of defects and their evolution (Minor & Kiener, 2011 ▸; Oh *et al.*, 2009 ▸; Kiener *et al.*, 2011 ▸; Minor *et al.*, 2001 ▸). However, electron-transparent samples are necessary for TEM investigations, whose preparation usually involves thinning of the sample, for instance by FIB milling. These additional sample preparation steps may alter the specimen microstructure and, thus, affect its mechanical behavior. In contrast, X-ray diffraction (XRD) techniques are non-invasive and do not require complicated sample preparations. Additionally, XRD is a very precise tool for recording strain with a resolution of 10^−4^ (Robinson & Harder, 2009 ▸; Newton *et al.*, 2010 ▸; Minkevich *et al.*, 2007 ▸) and for detecting defects, which introduce type-specific alterations in the diffraction pattern (Favre-Nicolin *et al.*, 2010 ▸; Jacques *et al.*, 2011 ▸). Therefore, *in situ* mechanical tests in combination with sub-micrometer focused X-ray diffraction methods have a great potential giving access to the strain as well as to defects, which are induced by the mechanical loading.

For instance, *in situ* µLaue diffraction studies on micrometer-sized structures in combination with compression or tensile tests using a micro-indenter have demonstrated the feasibility to identify the slip system activated during mechanical loading and to determine the density of geometrically necessary dislocations stored in the deformed material (Marichal *et al.*, 2013 ▸; Kirchlechner *et al.*, 2012 ▸; Maaß *et al.*, 2009 ▸). The alignment of the indenter head, the microstructure and the microfocused polychromatic X-ray beam was achieved by optical microscopy. However, the mutual alignment for nanosized objects and sub-micrometer focused beams is much more challenging making it necessary to image the nanostructured sample in combination with *in situ* X-ray techniques. Imaging with an indenter can only be performed in contact mode risking to damage or to induce plastic deformation in the nanostructures. Rodrigues *et al.* designed a special atomic force microscope (X-AFM) which was combined with a microfocused X-ray beam for local X-ray spectroscopy and *in situ* mechanical tests (Rodrigues *et al.*, 2008 ▸). Here, the mutual alignment was achieved by recording simultaneously a scanning X-ray diffraction map (SXDM) and a photocurrent image of the sample and the AFM-tip, respectively. Scheler *et al.* employed this X-AFM for studying the elastic deformation of a micrometric SiGe island during mechanical loading, while recording two-dimensional diffraction patterns (Scheler *et al.*, 2009 ▸). Beutier *et al.* combined the X-AFM with coherent X-ray diffraction to study the plastic deformation of a single copper island (Beutier *et al.*, 2013 ▸). This was further improved by Cornelius *et al.* by tuning the energy of the incident X-rays allowing for recording *in situ* the three-dimensional intensity distribution in reciprocal space around a given Bragg reflection (Cornelius *et al.*, 2012 ▸). However, this X-AFM contained an immobile AFM-tip complicating its alignment with respect to the X-ray beam. Additionally, the AFM-tips used in these experiments were hand-made electrochemically blunted tungsten wires, which were glued on one prong of a tuning fork. Each tip was unique, making comparison of two mechanical tests difficult. Also, the force applied on a selected structure could only be inferred from sophisticated modeling procedures taking several approximations into account (Rodrigues *et al.*, 2009 ▸).

In the current work, a new scanning force microscope for *in situ* nano­focused X-ray diffraction studies (SFINX) is presented. This new tool contains major improvements and advantages compared with the X-AFM as well as to micro- and nano-indenters. It allows for moving the sample and the AFM-tip independently facilitating any kind of alignment procedures. Additionally, commercial AFM cantilevers are used reducing the variability of mechanical tests. SFINX allows for *in situ* imaging of the sample topography and crystallinity by recording an AFM image in tapping mode and a scanning X-ray diffraction map (SXDM) simultaneously. This *in situ* imaging approach permits the perfect alignment of the tip, nanostructures, and a nanofocused X-ray beam with respect to each other. After the alignment, *in situ* mechanical tests can be performed. In the present paper, these capabilities of the new *in situ* device are exemplified on Au islands which were grown by a dewetting process on a sapphire substrate.

## 2. Experimental   

**Figure 1:**
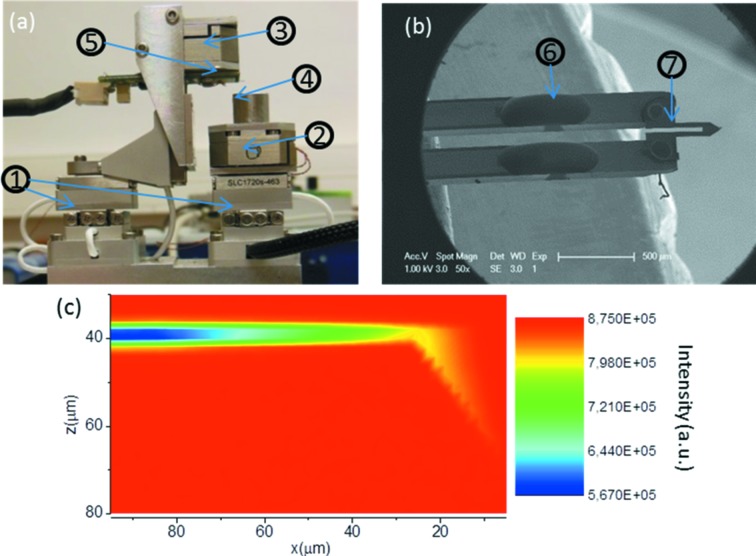
(*a*) Photograph of SFINX which contains (1) two stacks of long-range piezo stages, (2) *xy*-piezo scanner, (3) *z*-scanner, (4) sample stage and (5) AFM cantilever. (*b*) SEM image of the Akiyama probe consisting of (6) a quartz tuning fork and (7) a Si tip. (*c*) Scanning X-ray transmission map of the AFM tip.

The SFINX is presented in Fig. 1(*a*) ▸. It has been designed compact and light, measuring less than 10 cm × 10 cm × 10 cm and weighing less than 1 kg. This compactness facilitates its compatibility with different beamline endstations and, thus, with various X-ray techniques. SFINX consists of two stacks of long-range piezo stages [(1) in Fig. 1(*a*) ▸] with a stroke of 12 mm allowing for the mutual alignment of the AFM-tip and the sample with respect to a sub-micrometer focused X-ray beam. Furthermore, the lateral *xy* and the vertical *z* piezo scanners for AFM imaging were separated to improve the system stability [(2) and (3) in Fig. 1(*a* ▸)]. For reasons of compactness, a self-sensing cantilever, *i.e.* an Akiyama probe (provided by Nanoandmore) (Akiyama *et al.*, 2003 ▸), is employed. It consists of a quartz tuning fork (6) and a Si cantilever (7) with a stiffness of 5 N m^−1^ according to the provider (see Fig. 1*b* ▸). The resonance frequency and the quality factor (in air) of the probe are typically in the range 40–50 kHz and ∼1000, respectively. The influence of the cantilever on the X-ray beam is negligible as demonstrated by the X-ray scanning transmission map presented in Fig. 1(*c*) ▸ showing absorption of less than 2% close to the AFM-tip.

**Figure 2:**
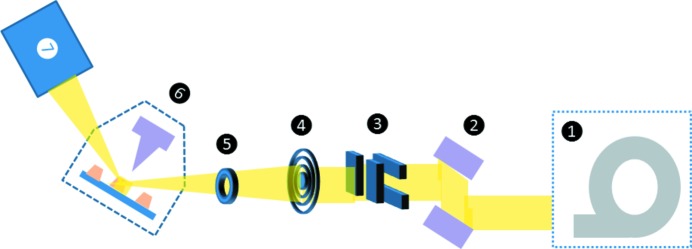
Schematic of the experimental set-up at the ID01 beamline at ESRF in Grenoble (France). (1) Synchrotron, (2) double-bounce channel-cut Si111 monochromator, (3) high-precision slits, (4) Fresnel zone plate, (5) order-sorting aperture, (6) SFINX, (7) detector: MAXIPIX or APD.

The new tool was installed on the diffractometer at the ID01 beamline at ESRF in Grenoble (France). The experimental set-up is schematically depicted in Fig. 2 ▸. During this experiment the X-ray beam was monochromated to an energy of 8.97 keV (λ = 0.138 nm) using the double-bounce channel-cut Si111 monochromator [(2) in Fig. 2 ▸]. The monochromator has a bandwidth of 10^−4^ which translates to a longitudinal coherence length of 1.4 µm. By means of a tungsten Fresnel zone plate (FZP) with a diameter of 300 µm and an outer zone width of 80 nm which was mounted in air, the monochromatic beam was focused down to 400 nm vertically (V) and 900 nm horizontally (H). The beam size was determined by a knife-edge scan employing a tungsten wire with diameter of 200 µm [(4) in Fig. 2 ▸]. An order-sorting aperture [(5) in Fig. 2 ▸] with a diameter of 50 µm was installed 2.5 cm upstream from the sample for selecting the first diffraction order of the FZP. High-precision slits [(3) in Fig. 2 ▸] were installed right in front of the FZP. For coherent X-ray diffraction studies, they were closed to match the transverse coherence lengths of the beamline amounting to approximately 20 and 60 µm in the horizontal and vertical direction, respectively. The diffractometer allows for rotating SFINX into the Bragg condition while keeping the AFM-tip always vertical with respect to the sample surface [(6) in Fig. 2 ▸]. When installed on a marble table in a standard laboratory in Marseille the peak-to-peak noise of SFINX amounts to ∼5 nm increasing to about 15 nm when being installed on the diffractometer. The difference in noise is attributed to the comparatively harsh environment at a synchrotron beamline where mechanical vacuum pumps run continuously and where the device is installed on a rotatable support of the diffractometer being less rigid than a marble table. The diffracted X-ray beam was recorded either by a two-dimensional MAXIPIX pixel detector with a pixel size of 55 µm × 55 µm or a point detector, *i.e.* an avalanche photodiode (APD) [(7) in Fig. 2 ▸]. Both detectors were mounted 1.27 m downstream from the sample position.

## 3. Results and discussion   

The potential of SFINX was explored on gold islands grown by dewetting a 20 nm thin gold film which was magnetron sputtered on a (0 0 0 1)-oriented sapphire substrate. While annealing the Au film at 1323 K it agglomerates on the substrate to form a large number of faceted Au islands which possess a flat (111) facet as upper surface, similar to the procedure published elsewhere (Sadan & Kaplan, 2006 ▸; Malyi *et al.*, 2011 ▸).

**Figure 3:**
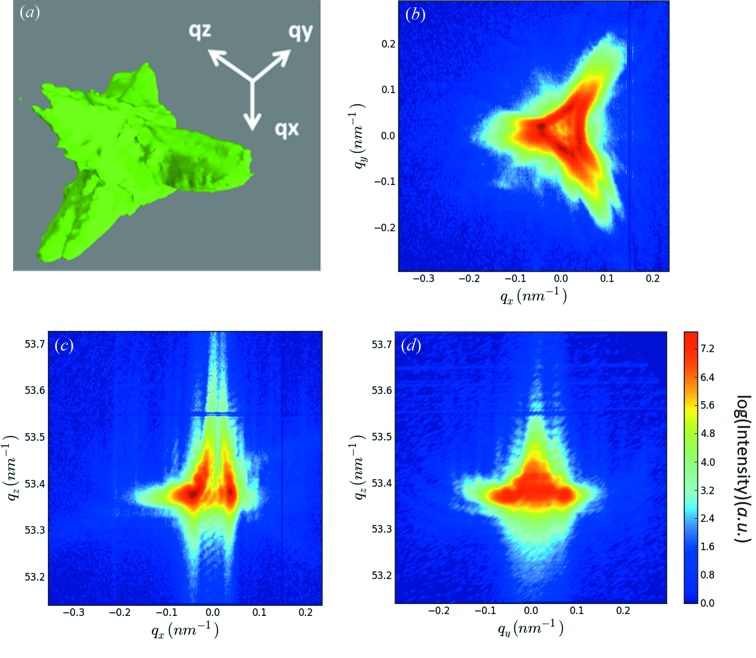
(*a*) Three-dimensional reciprocal space map in the vicinity of the Au222 Bragg reflection for a typical Au island and (*b*, *c*, *d*) two-dimensional cuts through the 3D-RSM shown in (*a*).

A coherent three-dimensional reciprocal space map (3D-RSM) of the Au222 Bragg reflection and the three corresponding two-dimensional cuts through reciprocal space for a typical Au island are shown in Figs. 3(*a*)–3(*d*) ▸. The 3D-RSM was recorded by rocking the sample ±0.5° (Δ*q* = ±0.47 nm^−1^, where *q* is the reciprocal space vector). The three-dimensional intensity distribution as well as the horizontal cut through the reciprocal space map (Fig. 3*b* ▸) shows a threefold symmetry spreading over ∼0.2 nm^−1^. It is centered at *q*_*z*_ = 53.42 nm^−1^ while the literature value for relaxed gold is *q*_*z*,Au(222)_ = 53.37 nm^−1^. This difference in *q*_*z*_ corresponds to a residual compressive out-of-plane strain of +0.09% within the island due to the dewetting process. The crystal truncation rod shows well defined size fringes from which the island height is inferred to be ∼265 nm.

### 3.1. *In situ* imaging   

**Figure 4:**
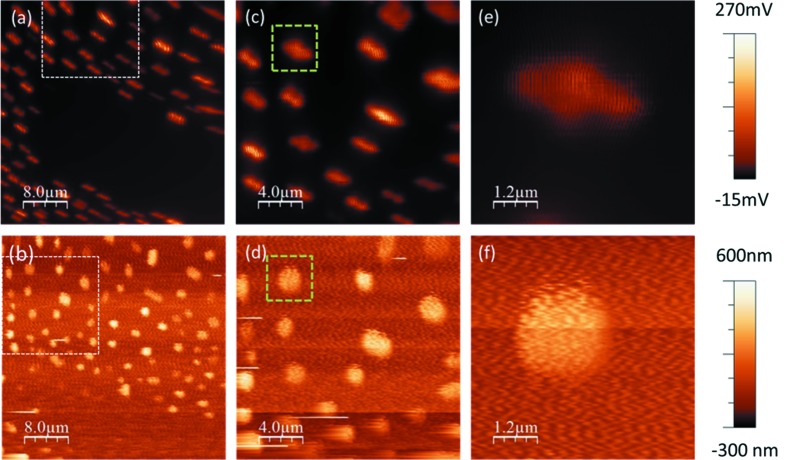
(*a*, *c*, *e*) Scanning X-ray diffraction maps and (*b*, *d*, *f*) simultaneously recorded AFM topography images of Au islands on sapphire substrate. The dashed squares in (*a*) and (*b*) mark the area imaged in (*c*) and (*d*) while the dotted squares in the latter images indicate the areas imaged in (*e*) and (*f*). All images were recorded at the Au222 Bragg reflection (θ = 36.20°).

For studying the properties of a certain nanostructure it has to be identified unambiguously. Therefore, first of all, the sample, the AFM-tip and the focused X-ray beam have to be aligned with respect to each other. By means of optical microscopy the AFM-tip and the zone of interest of the sample are adjusted with respect to the focal position of the X-ray beam with an accuracy of ∼10 µm. After this coarse alignment, the sample is scanned using the *xy* scanners [(2) in Fig. 1 ▸] while the sample topography is recorded with the AFM tip and, simultaneously, the diffracted X-rays are detected employing the avalanche photodiode. In order to increase the diffraction yield the FZP was fully illuminated and, thus, a partially coherent beam was used for the alignments. Figs. 4(*a*) and 4(*b*) ▸ show the *in situ* SXDM and the AFM image for Au islands which were recorded with a scan speed of 5 µm s^−1^ at the Au222 Bragg reflection (angle of incident beam θ = 36.20°). For AFM imaging a phase-locked loop was set up and a frequency shift of 10 Hz was maintained. The elongated shape of the island’s signal in the SXDM originates from the convolution of the island shape and the footprint of the X-ray beam which amounts to about 700 (V) nm × 900 (H) nm at the Bragg angle of 36.20°. When the two probes are perfectly aligned with respect to each other, both images, *i.e.* the SXDM and the topography, should show identical patterns. The comparison of the two *in situ* images reveals an offset of about 11 µm between the AFM-tip and the focused X-ray beam. The mutual alignment is improved by moving the tip using the long-range piezo stages of SFINX according to the determined offset. After compensation a second set of *in situ* images of a smaller area marked by the dashed square in Figs. 4(*a*) and 4(*b*) ▸ were recorded. The new pair of images displayed in Figs. 4(*c*) and 4(*d*) ▸ shows a diminution of the displacement of the AFM-tip with respect to the focused beam from 11 to about 1 µm. The iterative process of *in situ* imaging and offset compensation eventually results in a perfect alignment of the two probes as demonstrated by Figs. 4(*e*) and 4(*f*) ▸ presenting the same individual Au island selected for *in situ* study of its properties.

### 3.2. *In situ* indentation   

For *in situ* mechanical testing of a single nanostructure, the AFM-tip was positioned above a selected Au island, the feedback loop was switched off, and the nano-object was indented using the AFM-tip. Here, the tip was lowered in steps of 10 nm up to a total movement of 260 nm. Subsequently, it was raised likewise until the initial position was reached, and, finally, it was retracted by 5 µm. At each step a two-dimensional X-ray diffraction pattern was recorded employing the MAXIPIX detector. In order to reduce the acquisition time the FZP was fully illuminated and, thus, the X-ray beam was partially coherent. The exposure time amounted to 10 s.

**Figure 5:**
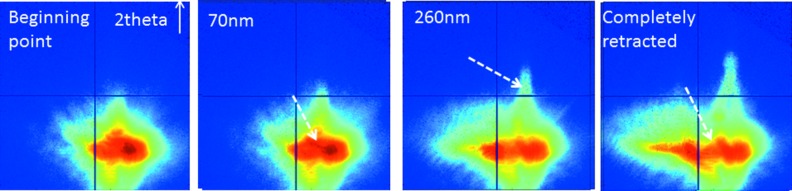
Sequence of *in situ* X-ray diffraction patterns recorded during the indentation process of an Au island. The dashed arrows in the images highlight the first changes observed during the indentation process, an elongation of the diffraction pattern along the 2θ-direction, and a spreading of the diffraction peak into several reflections.

A sequence of *in situ* diffraction patterns during the indentation test are displayed in Fig. 5 ▸. The initial diffraction pattern, when the AFM-tip is just above the top facet of the island, is displayed in the image on the left-hand side. The first change in the diffraction pattern was observed after lowering the AFM-tip by 70 nm as being highlighted by an arrow in the second image. When lowering the AFM-tip further and, thus, indenting the tip further into the Au crystal, the diffraction signal develops an inner structure and a streak along the 2θ direction appears. After retracting the tip completely, the diffraction pattern did not return to its initial shape evidencing a plastic deformation of the island.

For the time being, the force applied on a structure by means of the AFM-tip cannot be directly inferred. Within a first approximation the force is estimated assuming that the complete movement in the *z*-direction is converted into the deflection of the cantilever which has a stiffness of *k* = 5 N m^−1^ (according to the provider). Thus, the first changes observed in the diffraction pattern correspond to a force of roughly 350 nN. Molecular dynamic simulations (MD) on the indentation process on a Au (111) film employing a tip with a radius of 8 nm show a first pop-in event for an applied force of 300 nN (Kelchner *et al.*, 1998 ▸). Simulations on similar islands as studied in the present work considering an atomically sharp tip reveal the first dislocation nucleation for a force of around 100 nN (Mordehai *et al.*, 2011 ▸). The radius of curvature of the AFM-tip used in this experiment amounts to about 15 nm. Considering the stochastic nature of pop-ins, the experimental findings are in rather good agreement with the MD simulations. Thus, the first changes of the diffraction pattern may be attributed to plastic deformation. During elastic deformation, no change in the diffraction pattern has been observed. This might be due to the elastic strain induced by the tip up to the critical force being small compared with the initial strain induced by the interface (0.09%).

### 3.3. 3D-RSM   

**Figure 6:**
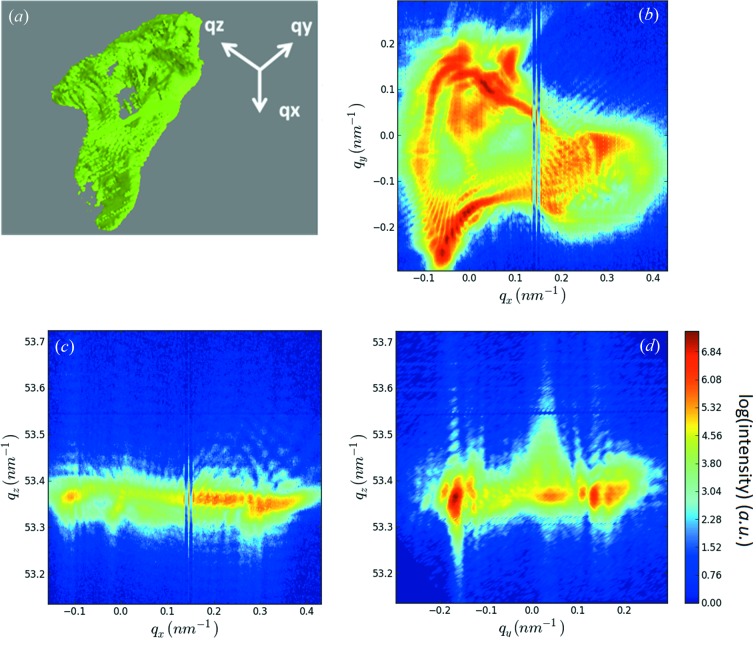
(*a*) Three-dimensional reciprocal space map in the vicinity of the Au222 Bragg reflection for the indented Au island shown in Figs. 3(*c*) and 3(*d*) ▸. (*b*, *c*, *d*) Two-dimensional cuts through the 3D-RSM shown in (*a*).

To shed additional light on the diffraction signal of the mechanically deformed Au islands a coherent three-dimensional reciprocal space map around the Au222 Bragg peak was recorded. For this purpose, the slits in front of the FZP were closed to match the lateral coherence lengths of the beamline and the sample was rocked by ±0.5° (Δ*q* = ±0.47 nm^−1^). The 3D-RSM and the three corresponding two-dimensional cuts through reciprocal space are presented in Figs. 6(*a*)–6(*d*) ▸. As in the case for the non-deformed island the 3D-RSM for the indented island shows a quasi-threefold symmetry (Fig. 3 ▸). However, the diffraction signal of the indented island covers about 0.5 nm^−1^ in the reciprocal space whereas the signal for the pristine structure spreads only over 0.2 nm^−1^. This increase in width in the lateral directions of the diffraction signal as well as the numerous speckles and fringes in the Bragg signal are evidence of the presence of both defects and strain induced by the mechanical loading.

The work presented here shows a great potential for the newly developed *in situ* atomic force microscope combined with sub-micrometer focused X-ray diffraction. This *in situ* technique allows for studying the onset of plasticity and the nucleation and evolution of defects induced by mechanical deformation. In the near future, SFINX will be installed in a vacuum chamber, thus minimizing a possible beam damage of the nanostructures. In addition, working under vacuum will also improve the long-term stability of the system which is necessary for employing coherent radiation and for recording *in situ* coherent 3D-RSM during mechanical deformation. *In situ* coherent diffraction will improve the sensitivity of this *in situ* technique and the measurement of the complete intensity distribution in the vicinity of a given Bragg reflection will eventually give direct access to the types of defects nucleated during mechanical loading. Furthermore, the *in situ* tool will be coupled in the near future with other sub-micrometer focused X-ray techniques such as µLaue diffraction and fluorescence mapping.

## 4. Conclusion   

In conclusion, the scanning force microscope for *in situ* nanofocused X-ray diffracton studies shows a great potential for *in situ* investigations of nanomaterials. It allows imaging simultaneously the sample topography and the crystallinity by recording an AFM image and a scanning X-ray diffraction map. In addition, it enables us to study the onset of plasticity in sub-micrometer structures as well as the evolution of defects during mechanical loading using the AFM-tip. The further combination of this tool with *in situ* three-dimensional reciprocal space mapping and coherent X-ray beams will improve the sensitivity of this *in situ* set-up for defects and, thus, the understanding of defect nucleation and evolution. The device may also be coupled with other X-ray techniques in the future such as X-ray fluorescence giving access to the elemental distribution on the nanoscale and µLaue diffraction which allows for determining the geometrically necessary dislocation density and various slip systems.

## References

[bb1] Akiyama, T., Staufer, U., de Rooij, N. F., Frederix, P. & Engel, A. (2003). *Rev. Sci. Instrum.* **74**, 112–117.

[bb2] Bei, H., Shim, S., George, E. P., Miller, M. K., Herbert, E. G. & Pharr, G. M. (2007). *Scr. Mater.* **57**, 397–400.

[bb3] Beutier, G., Verdier, M., Boissieu, M., d, Gilles, B., Livet, F., Richard, M.-I., Cornelius, T. W., Labat, S. & Thomas, O. (2013). *J. Phys. Conf. Ser.* **425**, 132003.

[bb4] Chen, C. Q., Shi, Y., Zhang, Y. S., Zhu, J. & Yan, Y. J. (2006). *Phys. Rev. Lett.* **96**, 075505. 10.1103/PhysRevLett.96.075505 16606107

[bb5] Cornelius, T. W., Davydok, A., Jacques, V. L. R., Grifone, R., Schülli, T., Richard, M.-I., Beutier, G., Verdier, M., Metzger, T. H., Pietsch, U. & Thomas, O. (2012). *J. Synchrotron Rad.* **19**, 688–694. 10.1107/S0909049512023758 22898946

[bb6] Favre-Nicolin, V., Mastropietro, F., Eymery, J., Camacho, D., Niquet, Y. M., Borg, B. M., Messing, M. E., Wernersson, L.-E., Algra, R. E., Bakkers, E. P. A. M., Metzger, T. H., Harder, R. & Robinson, I. K. (2010). *New J. Phys.* **12**, 035013.

[bb7] Jacques, V. L. R., Ravy, S., Le Bolloc’h, D., Pinsolle, E., Sauvage-Simkin, M. & Livet, F. (2011). *Phys. Rev. Lett.* **106**, 065502. 10.1103/PhysRevLett.106.065502 21405477

[bb8] Kelchner, C. L., Plimpton, S. J. & Hamilton, J. C. (1998). *Phys. Rev. B*, **58**, 11085–11088.

[bb9] Kiener, D., Grosinger, W., Dehm, G. & Pippan, R. (2008). *Acta Mater.* **56**, 580–592.

[bb10] Kiener, D., Hosemann, P., Maloy, S. A. & Minor, A. M. (2011). *Nat. Mater.* **10**, 608–613. 10.1038/nmat3055 PMC3145148 21706011

[bb11] Kirchlechner, C., Imrich, P. J., Grosinger, W., Kapp, M. W., Keckes, J. S., Micha, J., Ulrich, O., Thomas, O., Labat, S., Motz, C. & Dehm, G. (2012). *Acta Mater.* **60**, 1252–1258.

[bb12] Maaß, R., Petegem, S. V., Borca, C. N. & Swygenhoven, H. V. (2009). *Mater. Sci. Eng. A*, **524**, 40–45.

[bb13] Malyi, O., Klinger, L., Srolovitz, D. J. & Rabkin, E. (2011). *Acta Mater.* **59**, 2872–2881.

[bb14] Marichal, C., Van Swygenhoven, H., Van Petegem, S. & Borca, C. (2013). *Sci. Rep.* **3**, 2547. 10.1038/srep02547 PMC3757353 23989456

[bb15] Minkevich, A. A., Gailhanou, M., Micha, J.-S., Charlet, B., Chamard, V. & Thomas, O. (2007). *Phys. Rev. B*, **76**, 104106.

[bb16] Minor, A. M. & Kiener, D. (2011). *Acta Mater.* **59**, 1328–1337.

[bb17] Minor, A. M., Morris, J. W. & Stach, E. A. (2001). *Appl. Phys. Lett.* **79**, 1625.

[bb18] Mordehai, D., Kazakevich, M., Srolovitz, D. J. & Rabkin, E. (2011). *Acta Mater.* **59**, 2309–2321.

[bb19] Newton, M. C., Leake, S. J., Harder, R. & Robinson, I. K. (2010). *Nat. Mater.* **9**, 120–124. 10.1038/nmat2607 20023632

[bb20] Oh, S. H., Legros, M., Kiener, D. & Dehm, G. (2009). *Nat. Mater.* **8**, 95–100. 10.1038/nmat2370 19151703

[bb21] Rabier, J., Montagne, A., Wheeler, J. M., Demenet, J. L., Michler, J. & Ghisleni, R. (2013). *Phys. Status Solidi C*, **10**, 11–15.

[bb22] Richter, G., Hillerich, K., Gianola, D. S., Mönig, R., Kraft, O. & Volkert, C. A. (2009). *Nano Lett.* **9**, 3048–3052. 10.1021/nl9015107 19637912

[bb23] Robinson, I. & Harder, R. (2009). *Nat. Mater.* **8**, 291–298. 10.1038/nmat2400 19308088

[bb24] Rodrigues, M. S., Cornelius, T. W., Scheler, T., Mocuta, C., Malachias, A., Magalhaes-Paniago, R., Dhez, O., Comin, F., Metzger, T. H. & Chevrier, J. (2009). *J. Appl. Phys.* **106**, 103525.

[bb25] Rodrigues, M. S., Dhez, O., Denmat, S. L., Chevrier, J., Felici, R. & Comin, F. (2008). *J. Instrum.* **3**, 12004.

[bb26] Sadan, H. & Kaplan, W. D. (2006). *J. Mater. Sci.* **41**, 5099–5107.

[bb27] Scheler, T., Rodrigues, M., Cornelius, T. W., Mocuta, C., Malachias, A., Magalha~es-Paniago, R., Comin, F., Chevrier, J. & Metzger, T. H. (2009). *Appl. Phys. Lett.* **94**, 023109.

[bb28] Uchic, M. D., Dimiduk, D. M., Florando, J. N. & Nix, W. D. (2004). *Science*, **305**, 986–989. 10.1126/science.1098993 15310897

[bb29] Wang, L., Liu, P., Guan, P., Yang, M., Sun, J., Cheng, Y., Hirata, A., Zhang, Z., Ma, E., Chen, M. & Han, X. (2013). *Nat. Commun.* **4**, 2413. 10.1038/ncomms3413 PMC3778763 24022231

[bb30] Wheeler, J. M. & Michler, J. (2013). *Rev. Sci. Instrum.* **84**, 045103. 10.1063/1.4795829 23635228

